# Which brain lesions produce spasticity? An observational study on 45 stroke patients

**DOI:** 10.1371/journal.pone.0210038

**Published:** 2019-01-24

**Authors:** Kyoung Bo Lee, Bo Young Hong, Joon Sung Kim, Bomi Sul, Sang Cheol Yoon, Eun-Kyu Ji, Dong Baek Son, Byong Yong Hwang, Seong Hoon Lim

**Affiliations:** 1 Department of Rehabilitation Medicine, St. Vincent’s Hospital, College of Medicine, The Catholic University of Korea, Seoul, Republic of Korea; 2 Department of Physical Therapy, College of Public Health & Welfare, The Yongin University, Gyeonggi-do, Republic of Korea; University at Buffalo, UNITED STATES

## Abstract

Spasticity is an important barrier that can hinder the restoration of function in stroke patients. Although several studies have attempted to elucidate the relationship between brain lesions and spasticity, the effects of specific brain lesions on the development of spasticity remain unclear. Thus, the present study investigated the effects of stroke lesions on spasticity in stroke patients. The present retrospective longitudinal observational study assessed 45 stroke patients using the modified Ashworth Scale to measure muscle spasticity. Each patient was assessed four times: initially (within 2 weeks of stroke) and at 1, 3, and 6 months after the onset of stroke. Brain lesions were analyzed using voxel-based lesion symptom mapping (VLSM) with magnetic resonance imaging images. Spasticity developed to a certain degree within 3 months in most stroke patients with spasticity. The VLSM method with non-parametric mapping revealed that lesions in the superior corona radiata, posterior limb of the internal capsule, posterior corona radiata, thalamus, putamen, premotor cortex, and insula were associated with the development of upper-limb spasticity. Additionally, lesions of the superior corona radiata, posterior limb of the internal capsule, caudate nucleus, posterior corona radiata, thalamus, putamen, and external capsule were associated with the development of lower-limb spasticity. The present study identified several brain lesions that contributed to post-stroke spasticity. Specifically, the involvement of white matter tracts and the striatum influenced the development of spasticity in the upper and lower limbs of stroke patients. These results may be useful for planning rehabilitation strategies and for understanding the pathophysiology of spasticity in stroke patients.

## Introduction

Spasticity is defined as a motor disorder characterized by velocity-dependent increases in the tonic stretch reflex that result from hyperexcitability of the stretch reflex, one component of upper motor neuron syndrome[1]. Spasticity can have two types of effects on the functional outcomes of stroke patients; spasticity of the upper limbs is usually accompanied by disabilities and would be a target for restoration of function, whereas spasticity of the lower limbs may interfere with standing. Thus, brain lesions that produce spasticity should be investigated independently according to the involvement of the upper or lower limbs.

Although the reported prevalence of spasticity following stroke is highly variable, ranging from 4 to 42.6%[2–5], few studies have investigated the effects of brain lesions on spasticity in patients with stroke[6, 7]. Nonetheless, several studies have demonstrated how brain lesions can contribute to the development of spasticity in stroke patients. For example, studies from our research group have shown that damage to the anterior putamen and thalamus is related to a poor prognosis for upper-limb function in stroke patients[8, 9] and that lesions affecting the globus pallidus, putamen, and caudate nucleus are related to a poor prognosis for gait[8, 10]. However, the exact locations of brain lesions that produce spasticity have yet to be identified. Therefore, the present study aimed to investigate the roles that specific brain lesions play in the development of spasticity using lesion symptom mapping methods[11–13] that included analyses of brain magnetic resonance imaging (MRI) scans and clinical evaluations in patients with first-ever supratentorial strokes.

## Methods

### Study design and participants

The present study was a retrospective longitudinal observational clinical trial that included the data of 45 right-handed first-stroke patients, were confirmed by neurologic symptom and initial brain imaging studies as MRI or CT scan, recruited from a single inpatient/outpatient center from August 2016 to July 2017. All subjects had suffered supratentorial strokes and met the following criteria: 1) 20 < ages of subjects ≤ 80, 2) first unilateral stroke, 3) ability to follow verbal instructions, and 4) a Fugl–Meyer Assessment (FMA) score <60 for the upper extremities or <28 for the lower extremities[14].

The exclusion criteria were as follows: 1) a history of spinal cord injury, 2) a history of musculoskeletal injury or surgery, 3) a history of inflammatory arthritis or inflammatory myopathy, and 4) peripheral nervous system disease. Demographic and brain MRI data were collected from all subjects to evaluate spasticity in the upper and lower limbs[15]. Brain lesions and lesion sizes were evaluated using a high-resolution 3-T anatomical MRI system with a 5-mm slice thickness within 14 days of stroke.

Because the present study was an observational investigation of spasticity, the exact sample size was not calculated beforehand. However, the sample sizes of previous studies varied from 30 to 42[6–8]; thus, it was determined that the sample size for this study would be more than 42 subjects. A total of 64 patients were initially enrolled during the recruitment period, but 8 patients were excluded due to diabetic polyneuropathy, 2 were excluded for a subsequent attack within 6 months, and 9 were excluded due to attrition, such as transfer to another rehabilitation center at a distant location.

All subjects received physical and occupational therapies based on a neurodevelopmental treatment approach (physical therapy) and a task-orientated approach (occupational therapy). The rehabilitation program was initiated within 21 days after stroke onset (mean: 10.6 ± 6.7 days) for all subjects and continued for up to 6 months after onset. The program consisted of sessions that lasted 1–2 hours per day, 5 days per week and included both physical and occupational therapies;[9] the subjects also received speech therapy (ST) as needed. All interventions were primarily focused on using and strengthening the affected limb, basic mat activity, symmetric weight bearing and transfer activities, and gait training and were not performed exclusively for a specific purpose[9]. The study protocol was reviewed and approved by the Institutional Review Board of Catholic University, College of Medicine (Registry No. VC16RISE0205); the need for informed consent was waived by the board.

### Evaluation of spasticity

Spasticity was evaluated using the modified Ashworth scale(MAS)[15, 16] at the initial assessment (within 2 weeks) during enrollment and then at 1, 3, and 6 months post-stroke onset. The examiner obtained the total scores of the affected elbow flexor, elbow extensor, and wrist flexor for the upper extremity and the knee extensor, knee flexor, and ankle plantar flexor for the lower extremity. The MAS measures muscle tone as an indicator of spasticity using a scale from 0 (MAS 0) to 5 (MAS 4); a MAS score of 0 indicates no increase in muscle tone, and a MAS score of 4 indicates that the joint is rigid during flexion or extension. Thus, the total scores were estimated 0 to 15 for upper and lower extremity, respectively. Examinations of the upper and lower extremities in the present study were based on the positions previously proposed by Bohannon and Blackburn et al.[15, 16] If spasticity were severely increased and interfere with subjects’ function within the experimental period, the subjects had been taken proper treatment with botulinum toxin injection or medication. We assessed spasticity as just before treatment in these cases and maintained the scores throughout the experimental period.

### Lesion analysis

Lesion locations and sizes were assessed using MRIcron software (http://www.mricro.com/mricron). T2 images were co-registered with each participant’s T1 MRI, and then, the T1 and lesion maps were normalized to the Montreal Neurologic Institute (MNI) space using statistical parametric mapping (SPM). To increase the statistical power for identifying a lesion pattern that had a significant contribution to spasticity independent of hemispheric lateralization, all lesion maps were flipped onto the left hemisphere. Using the MRIcron software, all lesions were mapped onto slices of T2-weighted images that were co-registered to T1-weighted template MRI scans from the Montreal Neurological Institute (MNI)[11, 17]. The numbers of MRI voxels involved in each stroke lesion were calculated, and all lesions were traced by a trained image analyst and confirmed by an experienced physiatrist; specialist for neurorehabilitation, who was blind to all clinical data except for the side of hemiparesis.

For more accurate analyses, the origin of each image (coordinates: 0 × 0 × 0 mm) was reoriented such that it was located close to the anterior commissure and the volume-of-interest (VOI) images were transformed to the left hemisphere. To analyze the mutual lesion maps, segmentation and normalization were employed [10, 12]. We used MR automated- segment-normalize function of a plugin toolbox (http://www.mricro.com/clinical-toolbox/) to map into the stereotaxic space using the normalization algorithm provided by the SPM8 (http://www.fil.ion.ucl.ac.uk/spm/software/spm8) software. All process were checked by experienced physiatrist (specialist for neurorehabilitation). A voxel-based lesion-symptom mapping (VLSM) procedure was developed to analyze the relationship between tissue damage and behavior on a voxel-by-voxel basis. In previous studies that investigated lesions contributing to severe spasticity[7, 18], VLSM was performed using binary data (with/without a deficit); thus, a cutoff had to be applied. However, in that approach, information that reflects varying degrees of spasticity may be lost[13]. To avoid this potential issue, a direct statistical comparison of lesions was performed according to the degree of spasticity in the upper and lower limbs using a VLSM method implemented in non-parametric mapping (NPM) software included in the MRIcron software[11]. Only voxels that exhibited lesions in at least 10% (n = 4) of all patients were included in the final analysis. Non-parametric Brunner–Munzel tests for continuous data were used[11] because the clinical deficit data were continuous. In the NPM analyses, a lower value refers to a poorer performance; thus, subjects with a score of 0 had more severe spasticity than did those with a score of 4. Hence, inverse coding was used to transform all scores so that higher values were indicative of lower muscle tone. Colored VLSM maps representing the *z* statistics were generated and overlaid onto the automated anatomical labeling (AAL) and Johns Hopkins University white matter templates provided with the MRIcron software[11]. P-values <0.05 were considered to indicate statistical significance.

### Statistical analysis

All data from the complete set of assessments were analyzed using SPSS software version 12.0 (SPSS, Inc.; Chicago, IL, USA). Chi-square (χ^2^) and t-tests were conducted to analyze demographic data, and two-way repeated-measures analysis of variance (ANOVA) tests were carried out to investigate changes in the upper and lower extremities (extremity × time change). Post-hoc analyses using the Bonferroni method were conducted with a level of significance set at P < 0.05.

## Results

The present study analyzed 45 patients (mean age: 57.2 ± 12.6 years; 22 women and 23 men). Of these patients, 19 had left hemiplegia and 26 right hemiplegia, and the mean lesion volume was 60587.13 ± 72617.56 voxels (Table 1). The stroke lesion location and volumes for each all subjects were demonstrated in S1 Table.

**Table 1:** Demographic data of the participants.

Demographics (n = 45)	
Sex, M/F (%)	48.9/51.1
Age	57.2 ± 12.6
Handedness, R/L (%)	100/0
Side of weakness, R/L (%)	57.8/42.2
Time from stroke to rehab, days^a^	10.6 ± 6.7
Stroke pathology, hemorrhage/infarction (%)	44.4/55.6
Neglect (%)	20.0
Brain injury location, n (%)	
Cortex	3 (6.7)
Subcortex	24 (53.3)
Mixed cortex & subcortex	18 (40.0)
Lesion volume voxels (n) ^a^	60587.13 ± 72617.56

^a^Mean ± SD.

M: male; F: female; R: right; L: left

**Fig 1:**
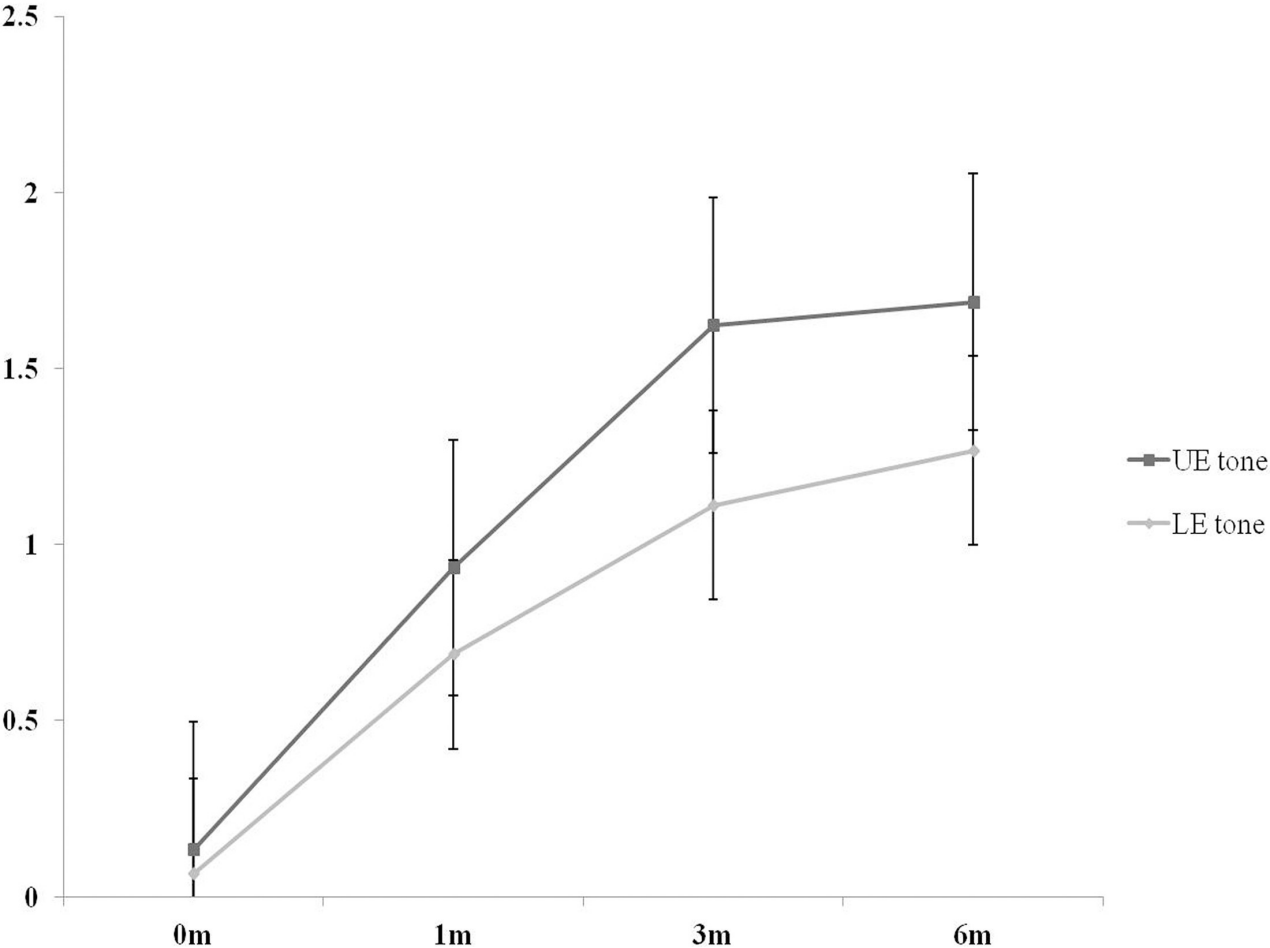
Comparisons of spasticity following stroke. The spasticity scores were increased significantly between initial and 3months, were not different between 3months and 6months after onset(p<0.05, Fig 1). The interaction between spasticity of upper limb and lower limb with time, was not different (p = 0.373). UE, upper extremity; LE, lower extremity.

All demographic data are shown in Table 1, and the development of spasticity as assessed by MAS over time is shown in Fig 1. Spasticity scores significantly increased between the initial assessment and 3 months but did not differ between 3 and 6 months after onset (p < 0.05, Fig 1). The interaction between spasticity of the upper and lower limbs with time was not significant (p = 0.373). The distributions of spasticity for the upper and lower limbs over time for all patients are shown in S2 and S3 Tables.

**Fig 2:**
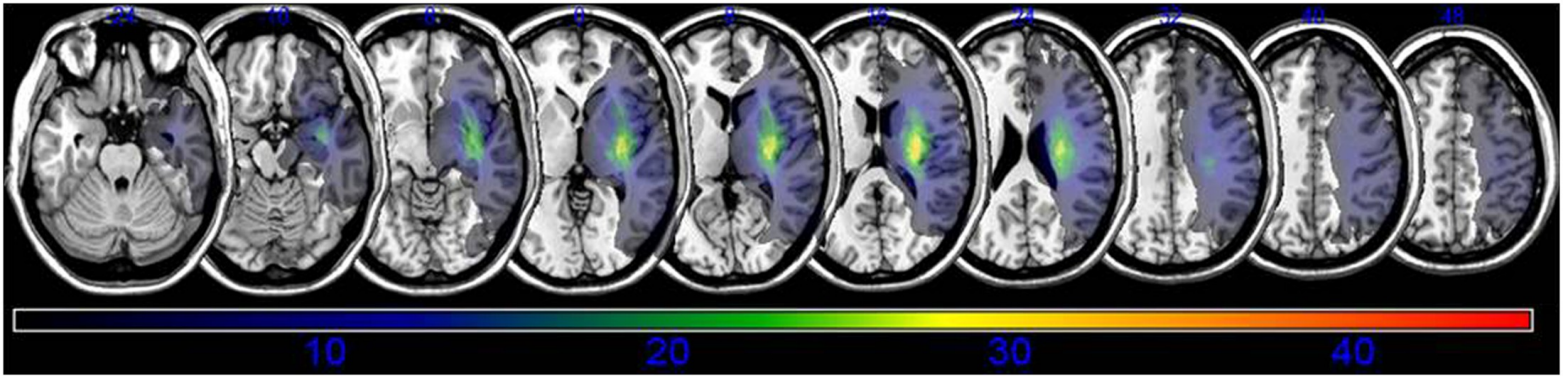
Overlay of lesions in all the subjects with stroke (*n* = 45). The color indicates the frequency of overlap.

**Fig 3:**
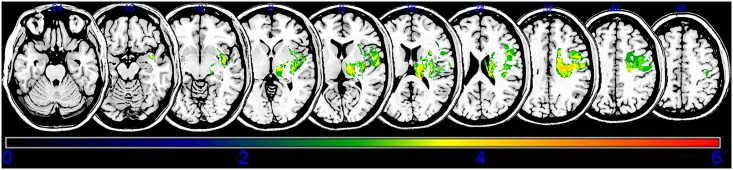
Statistical voxel-based lesion-symptom mapping for upper limb spasticity. The nonparametric Brubber Munzel statistical analysis was used for the continuous severe poststroke upper limb spasticity. Color scale indicates Brunner–Munzel rank order z-statistics. Only voxels significant at P<0.05 are shown. Colored bar represents the z statistics. The statistical map is displaying voxels with a minimum Z score of 2.4083. This matches the false discovery rate threshold. We set the maximum range of the Z score as 6, which be shown as being the maximum brightness.

**Fig 4:**
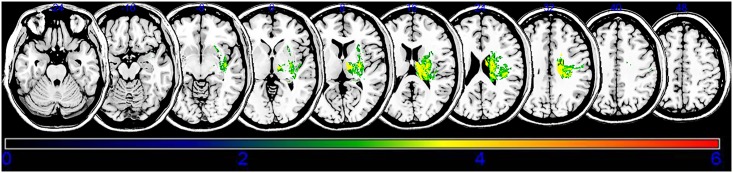
Statistical voxel-based lesion-symptom mapping for lower limb spasticity. The nonparametric Brubber Munzel statistical analysis was used for the continuous severe poststroke lower limb spasticity. Color scale indicates Brunner–Munzel rank order z-statistics. Only voxels significant at P<0.05 are shown. Colored bar represents the z statistics. The statistical map is displaying voxels with a minimum Z score of 2.5742. This matches the false discovery rate threshold. We set the maximum range of the Z score as 6, which be shown as being the maximum brightness.

An overlay of the lesions for all subjects is presented in Fig 2. The overlapping lesions of the brain for subjects without spasticity are shown in S1 and S2 Figs The VLSM method with NPM revealed that lesions of the superior corona radiata, internal capsule posterior limb, posterior corona radiata, thalamus, putamen, premotor cortex, and insula were associated with spasticity in the upper limbs (Fig 3, Table 2), whereas lesions of the superior corona radiata, internal capsule posterior limb, caudate nucleus, posterior corona radiate, thalamus, putamen, and external capsule were associated with spasticity in the lower limbs (Fig 4, Table 3).

**Table 2:** Stroke lesions related to upper extremity spasticity.

MNI coordinates (X, Y, Z)	BM Z max	n Voxels	Anatomical brain lesion
26, -19, 34	3.89059	108	Superior corona radiata
14, -11, 14	3.89059	107	Posterior limb
21, -25, 27	3.19465	96	Posterior corona radiata
18, -19, 6	3.89059	105	Thalamus
27, -8, 4	4.33277	98	Putamen
46, 0, 33	3.43161	110	Premotor cortex
40, -3, -8	3.61606	79	Insula

Montreal Neurological Institute coordinates representing voxels that were found significant based on the Brunner–Munzel Z score and the number (*n*) of clustering voxels that survived the false discovery rate-corrected threshold of *p* < 0.05. Anatomical regions are identified using the automated anatomical labeling (AAL) and the Johns Hopkins University (JHU) white matter templates.

**Table 3:** Stroke lesions related to lower extremity spasticity.

MNI coordinates (X, Y, Z)	BM Z max	n Voxels	Anatomical brain lesion
21, -10, 23	3.45575	112	Superior corona radiata
22, -14, 8	4.42564	109	Posterior limb
20, -7, 25	3.61530	112	Caudate nucleus
26, -26, 31	3.61530	113	Posterior corona radiata
12, -14, 8	3.89059	95	Thalamus
28, -8, 9	3.61446	100	Putamen
32, -7, 5	3.48003	101	External capsule

Montreal Neurological Institute coordinates representing voxels that were found significant based on the Brunner–Munzel Z score and the number (*n*) of clustering voxels that survived the false discovery rate-corrected threshold of *p* < 0.05. Anatomical regions are identified using the automated anatomical labeling (AAL) and the Johns Hopkins University (JHU) white matter templates.

## Discussion

Although previous studies have demonstrated that several general brain lesion locations are related to spasticity in stroke patients[7], the effects of specific brain lesions on the development of spasticity for the upper or lower limbs upper limb remain unclear. Here, we investigated the effects of stroke lesions on developing spasticity for the upper or lower limbs upper limb, respectively, in patients with stroke using VLSM. Our results suggest that involvement of the superior corona radiate, internal capsule posterior limb, posterior corona radiate, thalamus, putamen, premotor cortex and insula were related with spasticity of upper limb. The superior corona radiate, internal capsule posterior limb, caudate nucleus, posterior corona radiate, thalamus, putamen and external capsule were related with spasticity for lower limb. The previous researches showed that putamen, internal capsule posterior limb, external capsule, thalamus, and insula were associated with spasticity of upper limbs in patients with stroke [7, 18]. Another research showed the association of post-stroke spasticity with the insula, basal ganglia, thalamus, and white matter tracts [6]. Our results support previous reports [6, 7, 18]. In addition, the premotor cortex involved in developing spasticity of the upper limb. The caudate nucleus and external capsule were proved for association with developing spasticity of the lower limb.

More specifically, damage to the insula was associated with the development of spasticity in the upper limbs. The insula may play a role in vestibular function[19] and, damage in this area might therefore cause disturbances in the vestibulospinal system and contribute to spasticity of an upper limb[20]. The present results demonstrated that the superior corona radiata, internal capsule posterior limb, and posterior corona radiata were also associated with developing spasticity. The corticospinal system exerts inhibitory effects on spinal reflexes[21], and it is possible that the disruption of white matter pathways, such as the corona radiata and internal capsule, could alter inhibition. Damage to the premotor cortex, which is known as the origin of the corticoreticular pathway[22, 23], has been associated with the development of spasticity in the upper limbs, and it has been shown that this type of damage can have differential inhibitory and excitatory effects at the spinal level. In other words, if neural inhibitory systems were damaged by a stroke, then the remaining excitatory systems would be more active and alter behavioral functions[20]. Thus, damage to the premotor cortex likely contributed to spasticity in the upper limbs in stroke patients in the present study.

Damage to the putamen and thalamus may also affect spasticity of the upper limbs. For example, the insula, thalamus, basal ganglia, and white matter tracts are significantly associated with severe upper limb spasticity in post-stroke patients[7]. Additionally, other studies have shown that damage to the anterior putamen and thalamus plays roles in selective and isolated motor control of the upper limb[8, 9, 24, 25]. Taken together with the present results, these previous findings indicate that the putamen and thalamus are involved in the control of muscle tone and contribute to fine adjustments in the upper limbs[9, 25, 26].

Damage to the caudate nucleus, putamen, and thalamus is also related to spasticity in the lower limbs of stroke patients. Brain lesions associated with spasticity in the lower limbs have yet to be fully elucidated, although some previous studies have shown that the corona radiata, internal capsule, globus pallidus, putamen, cingulum, primary motor cortex, and caudate nucleus play roles in the recovery of gait[8, 10]. Of these areas, the striatum is a pattern generator for gait[27, 28], and damage to the posterolateral putamen is associated with temporal gait asymmetry[28]. Furthermore, neuronal injury in the corona radiata, caudate nucleus, and putamen of patients with chronic stroke changes walking speed[7, 29] and functional connectivity within the striatum influences lower-limb functions, particularly gait[30, 31]. Taken together, these findings suggest that the striatum and thalamus play roles in lower-limb spasticity via the thalamo-striatal system, which is similar to the dysfunction observed in Parkinson’s disease and dystonia[32–34]. Thus, the striatum and thalamus likely play important roles in the development of spasticity in the upper and lower limbs. The present results support previous findings regarding patients with stroke, Parkinson’s disease, and dystonia[7, 8, 26, 27, 30, 33, 35] and may be useful and effective for the development and application of rehabilitiation strategies for these disorders.

Because the present study was not a large-scale investigation, several focusing methods were used to overcome biases. For example, only patients with supratentorial lesions and moderate to severe hemiplegia were included. In addition, subjects with hemorrhagic stroke were excluded initially, for overcome of some bias on calculating brain lesions. If treatment needed, we treated the spasticity with injection or medication. These biases were un-avoidable in the retrospective design. We assessed the spasticity as the worst status of the subject (just before treatment). Thus, we did not evaluate the natural remission of spasticity. Lastly, we used the method for VLSM with flipping onto the left hemisphere. The flipping might increase statistical power. In addition, until now, there is no evidence of the effect of laterality on spasticity. However, there is a possibility of uncovering bias which laterality may intervene in developing spasticity.

The present results demonstrated the involvement of the superior corona radiata, internal capsule posterior limb, posterior corona radiata, thalamus, putamen, premotor cortex, and insula in spasticity of the upper limbs and damage to the superior corona radiata, internal capsule posterior limb, caudate nucleus, posterior corona radiata, thalamus, putamen, and external capsule with spasticity of the lower limbs in patients with stroke. However, the present study was not able to reveal the functional brain networks underlying spasticity or a specific tract between the striatum and the cerebral cortex. Further research investigating these types of functional networks will be needed to address these issues.

## Conclusion

The involvement of white matter tracts and the striatum influences the development of spasticity in the upper and lower limbs of patients with stroke. These results may be useful for planning rehabilitation strategies and understanding the pathophysiology of spasticity in stroke patients.

## Supporting information

S1 Fig Overlay of lesions in all the subjects without spasticity of upper limbs at 6 months after onset (*n* = 18). The color indicates the frequency of overlap.
(TIF)

S2 Fig Overlay of lesions in all the subjects without spasticity of lower limbs at 6 months after onset (*n* = 25). The color indicates the frequency of overlap.
(TIF)

S1 Table The characteristics of stroke in all subjects. (DOCX)

S2 Table Distributions of the sum for muscle tone in upper extremity after stroke. (DOCX)

S3 Table Distributions of the sum for muscle tone in lower extremity after stroke. (DOCX)
