# Supplementary figures and images for: Which brain lesions produce spasticity? An observational study on 45 stroke patients
Source: PLoS One. 2019 Jan 24;14(1):e0210038. doi: 10.1371/journal.pone.0210038 (PMC6345431; doi:10.1371/journal.pone.0210038)

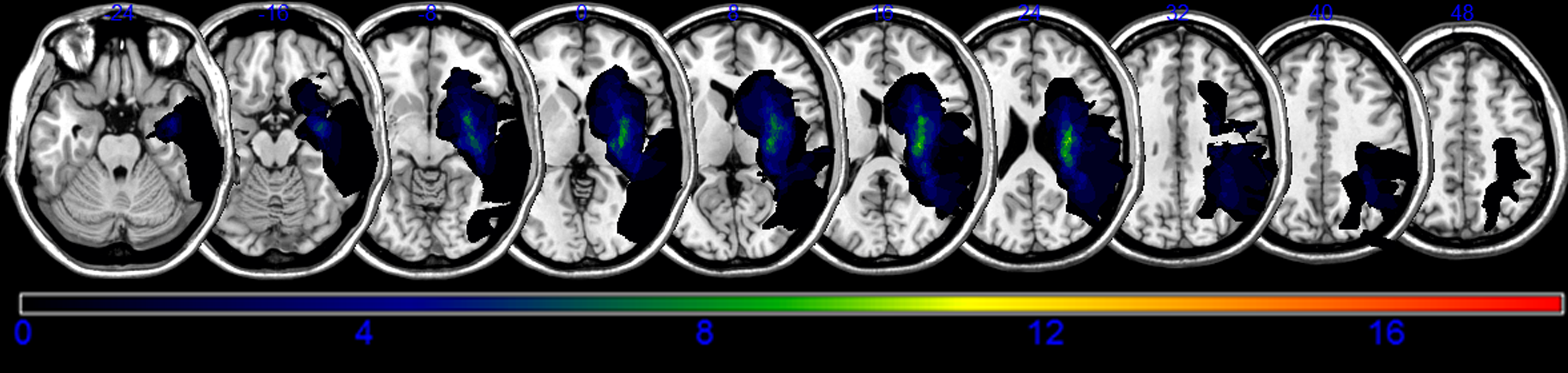

Supplement: S1 Fig — The color indicates the frequency of overlap. (TIF) [file pone.0210038.s001.tif]

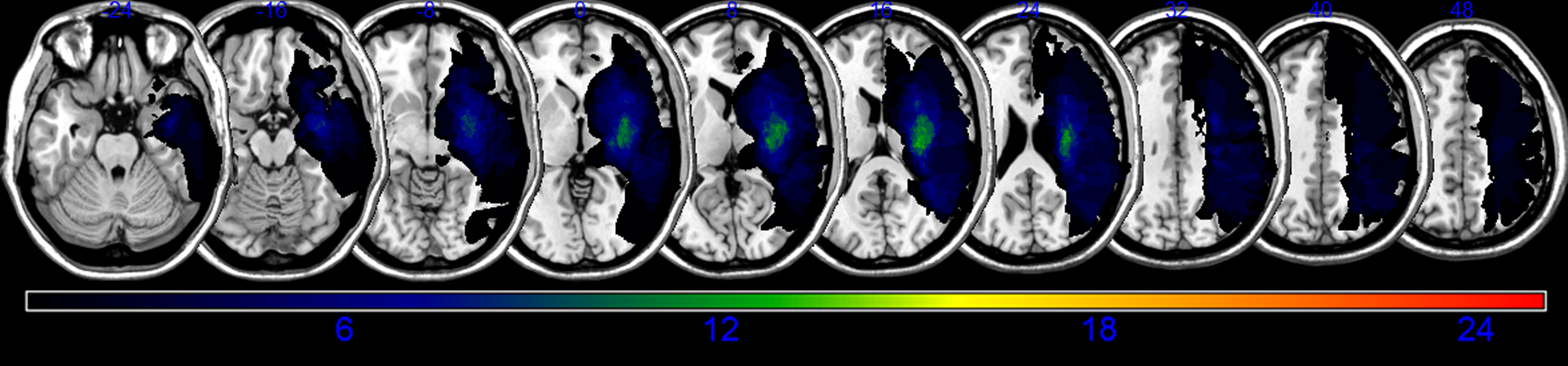

Supplement: S2 Fig — The color indicates the frequency of overlap. (TIF) [file pone.0210038.s002.tif]
