# Supplementary material for: Which brain lesions produce spasticity? An observational study on 45 stroke patients
Source: PLoS One. 2019 Jan 24;14(1):e0210038. doi: 10.1371/journal.pone.0210038 (PMC6345431; doi:10.1371/journal.pone.0210038)
Supplement: S1 Table — (DOCX) [file pone.0210038.s003.docx]

S1 Table. The characteristics of stroke in all subjects

| Number | Range of Age | Side of Paralysis | Type of Stroke | Location of Lesion | Volume of Stroke (Voxel) |
| --- | --- | --- | --- | --- | --- |
| 1 | 51-60 | Rt | Hemorrhagic | Mixed | 139845 |
| 2 | 61-70 | Lt | Hemorrhagic | Subcortex | 45191 |
| 3 | 71-80 | Lt | Ischemic | Cortex | 116576 |
| 4 | 71-80 | Rt | Hemorrhagic | Subcortex | 2111 |
| 5 | 61-70 | Lt | Ischemic | Cortex | 181823 |
| 6 | 41-50 | Lt | Ischemic | Mixed | 47701 |
| 7 | 61-70 | Rt | Hemorrhagic | Mixed | 47834 |
| 8 | 41-50 | Rt | Hemorrhagic | Subcortex | 46478 |
| 9 | 51-60 | Rt | Hemorrhagic | Subcortex | 9714 |
| 10 | 31-40 | Lt | Hemorrhagic | Mixed | 28783 |
| 11 | 51-60 | Lt | Hemorrhagic | Subcortex | 38154 |
| 12 | 71-80 | Rt | Ischemic | Subcortex | 2662 |
| 13 | 41-50 | Rt | Ischemic | Subcortex | 3148 |
| 14 | 21-30 | Rt | Hemorrhagic | Mixed | 128525 |
| 15 | 51-60 | Lt | Hemorrhagic | Subcortex | 100685 |
| 16 | 61-70 | Rt | Hemorrhagic | Subcortex | 5506 |
| 17 | 41-50 | Lt | Hemorrhagic | Mixed | 122367 |
| 18 | 41-50 | Lt | Ischemic | Mixed | 115139 |
| 19 | 51-60 | Lt | Hemorrhagic | Mixed | 82402 |
| 20 | 41-50 | Lt | Ischemic | Mixed | 326250 |
| 21 | 51-60 | Rt | Hemorrhagic | Subcortex | 25327 |
| 22 | 61-70 | Rt | Hemorrhagic | Subcortex | 63579 |
| 23 | 51-60 | Rt | Hemorrhagic | Subcortex | 26647 |
| 24 | 41-50 | Rt | Hemorrhagic | Subcortex | 90617 |
| 25 | 61-70 | Lt | Ischemic | Subcortex | 37293 |
| 26 | 41-50 | Rt | Hemorrhagic | Subcortex | 20870 |
| 27 | 71-80 | Lt | Ischemic | Subcortex | 9492 |
| 28 | 51-60 | Rt | Hemorrhagic | Subcortex | 16709 |
| 29 | 51-60 | Lt | Hemorrhagic | Mixed | 77986 |
| 30 | 61-70 | Lt | Ischemic | Mixed | 30444 |
| 31 | 61-70 | Lt | Ischemic | Subcortex | 6154 |
| 32 | 61-70 | Rt | Ischemic | Cortex | 142055 |
| 33 | 61-70 | Lt | Ischemic | Mixed | 294766 |
| 34 | 71-80 | Rt | Ischemic | Mixed | 7622 |
| 35 | 41-50 | Rt | Ischemic | Mixed | 97847 |
| 36 | 51-60 | Rt | Ischemic | Mixed | 12923 |
| 37 | 51-60 | Lt | Ischemic | Mixed | 4860 |
| 38 | 61-70 | Rt | Ischemic | Subcortex | 535 |
| 39 | 41-50 | Rt | Ischemic | Subcortex | 4604 |
| 40 | 51-60 | Lt | Ischemic | Mixed | 102488 |
| 41 | 61-70 | Rt | Ischemic | Subcortex | 4507 |
| 42 | 51-60 | Rt | Ischemic | Mixed | 22372 |
| 43 | 51-60 | Rt | Ischemic | Subcortex | 5307 |
| 44 | 51-60 | Rt | Ischemic | Subcortex | 832 |
| 45 | 71-80 | Rt | Hemorrhagic | Subcortex | 29691 |

Mixed: Cortex and Subcortex.
