# Supplementary material for: Which brain lesions produce spasticity? An observational study on 45 stroke patients
Source: PLoS One. 2019 Jan 24;14(1):e0210038. doi: 10.1371/journal.pone.0210038 (PMC6345431; doi:10.1371/journal.pone.0210038)
Supplement: S2 Table — (DOCX) [file pone.0210038.s004.docx]

S2 Table. Distributions of the sum for muscle tone in upper extremity after stroke

| UE Muscle tone | 0 month | 1 month | 3 months | 6 months |
| --- | --- | --- | --- | --- |
| MAS 0 | 39 | 24 | 19 | 18 |
| MAS 1 | 6 | 7 | 5 | 5 |
| MAS 2 |  | 8 | 6 | 8 |
| MAS 3 |  | 5 | 9 | 7 |
| MAS 4 |  | 1 | 3 | 5 |
| MAS 5 |  |  | 1 | 1 |
| MAS 6 |  |  | 2 | 1 |

UE, upper extremity; MAS, modified Ashworth scale. The sum of muscle tone was obtained by affected elbow flexor, elbow extensor, and wrist flexor.
