# Supplementary material for: Which brain lesions produce spasticity? An observational study on 45 stroke patients
Source: PLoS One. 2019 Jan 24;14(1):e0210038. doi: 10.1371/journal.pone.0210038 (PMC6345431; doi:10.1371/journal.pone.0210038)
Supplement: S3 Table — (DOCX) [file pone.0210038.s005.docx]

S3 Table. Distributions of the sum for muscle tone in lower extremity after stroke

| LE Muscle tone | 0 month | 1 month | 3 months | 6 months |
| --- | --- | --- | --- | --- |
| MAS 0 | 42 | 26 | 24 | 25 |
| MAS 1 | 3 | 11 | 9 | 8 |
| MAS 2 |  | 4 | 3 | 1 |
| MAS 3 |  | 4 | 5 | 5 |
| MAS 4 |  |  | 2 | 2 |
| MAS 5 |  |  |  | 1 |
| MAS 6 |  |  | 2 | 3 |

LE, lower extremity; MAS, modified Ashworth scale. The sum of muscle tone was obtained by affected knee extensor, knee flexor, and ankle plantar flexor.
